# Pemphigus relapse: Mechanisms, risk factors, and agents associated with disease recurrence

**DOI:** 10.1111/1346-8138.17505

**Published:** 2024-10-26

**Authors:** Gaurav N. Pathak, Priya Agarwal, Sydney M. Wolfe, Kush H. Patel, Jimmy Dhillon, Babar K. Rao

**Affiliations:** ^1^ Department of Dermatology Rutgers Robert Wood Johnson Medical School Somerset New Jersey USA; ^2^ Department of Dermatology Rao Dermatology Atlantic Highlands New Jersey USA

**Keywords:** pemphigus, pemphigus foliaceus, pemphigus vulgaris, relapse, rituximab

## Abstract

Pemphigus represents a spectrum of potentially life‐threatening autoimmune‐mediated skin blistering conditions caused by antibody production against desmoglein 1 and 3 (anti‐DSG 1 and 3) in keratinocytes. Greater than 50% of pemphigus patients experience relapse, which complicates long‐term medical management, including risks associated with re‐treatment and complications such as infection and dehydration. This review aims to elucidate mechanisms, risk factors, and medications associated with pemphigus relapse. Mechanisms of relapse include the persistence of auto‐reactive B‐cell populations post‐treatment and CD20‐ B‐cell populations that reactivate after B‐cell depletion therapy. Risk factors for relapse include high body surface area (BSA) of pemphigus involvement, high body mass index, high severity according to the Pemphigus Disease Area Index (PDAI) at onset, treatment delay, and high anti‐DSG1 and DSG3 titers post‐treatment. Targeted B‐cell localization is associated with better clinical outcomes, including less frequent relapses. Rituximab is currently the gold standard of treatment for moderate–severe pemphigus and has relapse rates of 11%–44% in selected studies, with a mean time to relapse of 5.8 months to 36 months following treatment. Relapse rates across lymphoma dosing (375 mg/m^2^) versus rheumatoid arthritis dosing (1 g dosing weekly) was inconsistent; however, more frequent dosing, earlier treatment, and higher cumulative dosing were associated with lower relapse rates. Alternative agents that have clinical efficacy include corticosteroid monotherapy, mycophenolate mofetil, azathioprine, and intravenous immunoglobulin. Future studies should include head‐to‐head comparators over long follow‐up periods to identify the best treatment agents associated with the least relapse risk.

## 1 INTRODUCTION

Pemphigus is a spectrum of rare autoimmune diseases characterized by intra‐epithelial blistering of the skin and mucosal membranes. Pemphigus is caused by autoantibody production against desmoglein 1 and 3 (anti‐DSG1 and DSG3), which are involved in the cell‐to‐cell adhesion of keratinocytes.^1^, ^2^ These conditions are associated with high morbidity and high mortality (1.6%–12%) with a worse prognosis in patients who are immunocompromised, over 65 years of age, and who have comorbid autoimmune disease or cardiovascular disease.^1^, ^3^, ^4^

The primary goal of therapy is to heal pre‐existing lesions while preventing the formation of new lesions. Initial treatment of pemphigus consists of systemic corticosteroids because of their rapid therapeutic effects. However, these regimens must eventually be tapered to avoid adverse effects (AEs) that are characteristic of long‐term steroid use, including weight gain, osteoporosis, and heightened infection risk.^1^ Rituximab, an anti‐CD20 monoclonal antibody, is the first agent to receive US Food and Drug Administration (FDA) approval for moderate to severe pemphigus vulgaris (PV), and has improved remission rates and long‐term clinical outcomes.^1^, ^2^

Pemphigus relapse following initial treatment poses a considerable challenge in long‐term disease management. Although there is no universal definition for relapse, it is generally defined as the appearance of three or more new lesions a month that do not heal spontaneously within 1 week, or by the extension of established lesions in a patient who has achieved disease control.^5^ More than half of pemphigus patients will experience relapse during their disease course increasing the burden of disease and negatively impacting patients' quality of life.^1^, ^2^, ^6^, ^7^ An in‐depth understanding of the mechanisms of resistance to treatment as well as risk factors and therapeutic agents associated with pemphigus relapse, may guide clinical decision‐making. This review aims to elucidate the clinical and immunological factors contributing to pemphigus relapse and identify interventions and risk factors to reduce relapse rates for pemphigus patients.

## 2 METHODS

A comprehensive literature search for studies involving mechanisms, risk factors, and interventions for pemphigus (with associated relapse rates) was performed on four separate databases: Medline (PubMed), Cochrane Library, Clinicaltrials.gov, and Embase. The search term for all three platforms was “pemphigus relapse risk.” The authors limited their search to publications of randomized clinical trials (RCTs), observational studies, case reports, case series, and relevant literature reviews that were published within the last 10 years. Each study must have evaluated some aspect of the mechanism, risk factor for, or relapse rate of different interventions to be eligible for the review. Eligible interventional studies included any type of relapse statistic including rate of relapse, duration of relapse, or time until relapse for a pemphigus indication. Exclusion criteria included studies not published in English, studies not evaluating relapse, those evaluating a non‐pemphigus condition (including Hailey‐Hailey disease), or articles lacking full free access.

## 3 RESULTS

### 3.1 Mechanisms of pemphigus relapse

Pemphigus develops due to the production of anti‐DSG1 and DSG3 antibodies by errant autoreactive B‐cells. B‐cell depleting agents, such as rituximab, are non‐selective CD20 inhibitors that reduce active autoreactive and active normal long‐lived B‐cell populations. Mechanisms of pemphigus relapse include both incomplete B‐cell depletion and the persistence of DSG3‐self‐reactive B‐cell populations that are notably present in patients who relapse but not in those experiencing clinical remission.^8^ Rituximab does not fully deplete B‐cells in secondary lymphoid organs and has poor absorption and permeation in skin, which may also contribute to relapse.^9^ Since epitope spreading is rarely found in pemphigus vulgaris (PV), B‐cells responsible for relapse are often the same cohort of autoreactive B‐cells that originally caused pemphigus.^10^

**FIGURE 1:**
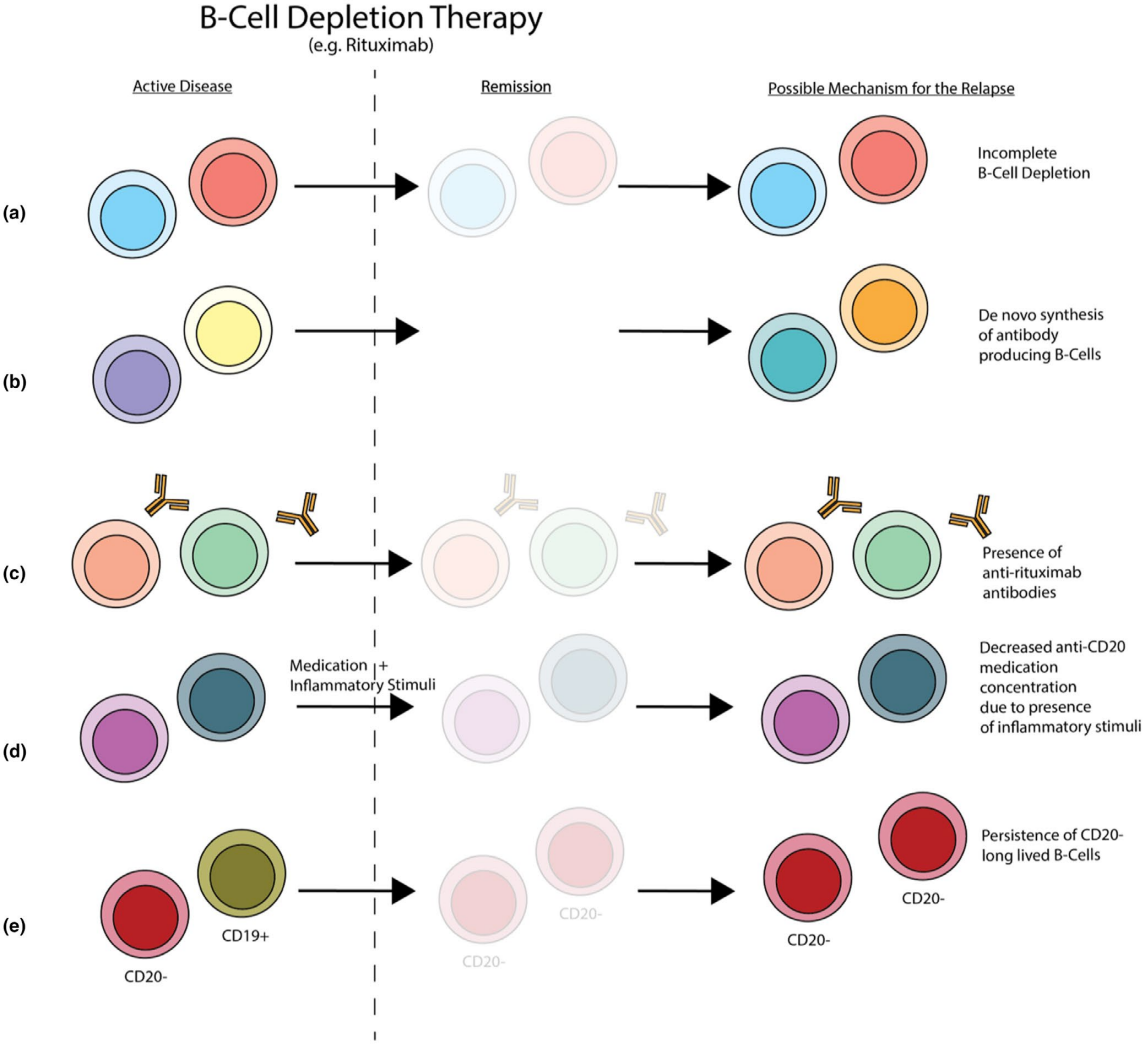
Mechanisms of pemphigus relapse post B‐cell depletion therapy. (A) Incomplete B‐cell depletion of autoantibody‐producing B‐cells. (B) De novo synthesis of new autoantibody‐producing B‐cells. (C) Presence of anti‐rituximab antibodies leading to incomplete B‐cell depletion. (D) Increased inflammatory stimuli reducing B‐cell depletion and leading to persistence of autoantibody‐producing B‐cells. (E) Persistence of long‐lived CD20‐ B‐cells that are not targeted by B‐cell depletion therapies. Some concepts of this illustration are adapted from Ellebrecht and Payne.^11^

Another possible mechanism of disease relapse is the de novo production of new anti‐DSG1 and DSG3 B‐cells after treatment. Patients with long‐term relapse, however, do not have the same anti‐DSG B‐cell auto‐reactive clones, suggesting that PV relapse is primarily due to non‐tolerant B‐cell lineages and not new autoreactive cells that occur frequently and escape tolerance mechanisms (Figure 1).^8^, ^9^

Rituximab may preferentially deplete activated CD19+ cells in the peripheral blood and bone marrow; however, re‐activation of anti‐DSG1 and DSG3 autoantibodies made by long‐lived CD20‐ plasma cells, which are not targeted by B‐cell depleting regimens, may lead to relapse.^12^ Because pemphigus conditions are associated with diagnostic delay, those who may not have received treatment until later stages of the disease may be at a higher risk for this mechanism of relapse (Figure 1).^11^, ^13^, ^14^ Other potential reasons for relapse are drug resistance factors including CD20 downregulation, genetic factors impairing antibody‐dependent cellular toxicity, and anti‐drug antibodies.^13^

### 3.2 Risk factors for relapse

Identification of patients who might be at a heightened risk of relapse may guide preventative, screening, and treatment efforts to optimize patients' time in clinical remission. Although relapse rates vary with treatment modality, patient characteristics, laboratory findings, and other biometrics have been found to predict therapeutic response and relapse.^6^

A study that included 143 pemphigus patients saw a higher risk of relapse for a baseline body surface area (BSA) score of 3 (>15% mucocutaneous involvement) compared with a BSA score of <3 (odds ratio [OR]  3.30, 95% confidence interval [CI] 1.17–9.28; *p* = 0.02).^15^ If there is a positive titer of either anti‐DSG1 or anti‐DSG3 autoantibodies at the conclusion of treatment, there is a higher risk of relapse (OR 2.42, 95% CI 1.21–4.85, *p* = 0.01). Other factors found to be significantly associated with early clinical relapse were a raised CD19+ B‐cell count at baseline (OR 7.84; 95% CI 1.39–53.41, *p* = 0.01), IgG (OR 4.85; 95% CI 1.09–23.44, *p* = 0.04), and C3 (OR 20.33; 95% CI 3.02–199.5, *p* < 0.001) positivity in the intercellular space of the epidermis on direct immunofluorescence.^16^

Positive testing of DSG1 (>20 IU) was predictive of relapse in mucocutaneous disease (hazard ratio [HR] 6.40, *p* = 0.19), whereas a positive test for DSG3 (>20 IU) was predictive of relapse (HR 32.92, *p* < 0.01) with, primarily, mucocutaneous and mucosal disease.^17^ Patients with CD4 counts of <400 cells/μL were approximately 25 times more likely to relapse compared to those with higher CD4 counts (*p* < 0.001), and every 200 cells/μL increase in CD4 count decreased the HR of relapse by 35% (*p* = 0.029).^17^

The Pemphigus Disease Area Index (PDAI) is a disease severity measurement tool for pemphigus patients based on the number and size of lesions.^18^ A PDAI score of >45 and/or persistent anti‐DSG1 (>20 IU/mL) or anti‐DSG3 (>130) at month 3 had a positive predictive value (PPV) of 50% (95% CI 27%–73%) and a negative predictive value (NPV) of 94% (95% CI 73%–100%) post B‐cell depleting treatment.^19^ Additionally, cytochrome c release from keratinocytes has been investigated as a predictive biomarker for pemphigus, however, it lacks a strong correlation with disease relapse.^20^ The presence of three or more anti‐DSG3 IgG subclasses was predictive of relapse (PPV 62.5%, and NPV 92%).^21^ Rarely, pemphigus patients may relapse without the detection of autoantibodies against DSG, which may be long‐lasting.^22^

Age >65 was identified as a predictive factor for complete remission of therapy, possibly due to weakened immune systems making reactivation of autoimmune conditions less likely.^23^ High body mass index (BMI) (>35) is a negative prognostic factor of achieving complete remission of pemphigus after 1 cycle.^23^ This may be due to inconsistent B‐cell depletion in weight‐based lymphoma protocol (LP) dosing strategies, pro‐inflammatory states in patients with high BMI, or impaired immune function in obese patients.^24^ Sex, pemphigus subtype (PV and pemphigus foliaceus), and total disease duration were not predictive of clinical relapse.^23^ However, mucosal subtypes of PV are associated with poor clinical outcomes following treatment and are associated with an increased risk of relapse (OR 4.63; *p* = 0.034).^25^

Age and number of months with disease did not correlate with 12, 18, or 24‐month relapse‐free scores (*p* = 0.41, *p* = 0.98, *p* = 0.61, respectively).^14^ Younger pemphigus patients appear to experience high rates of relapse, however, the small sample size and rarity of pediatric pemphigus limit the generalizability of these findings.^26^, ^27^ Other factors that have been associated with triggering pemphigus relapse in smaller scale studies include illicit drug use (cocaine) and viral reactivation (by COVID‐19, herpes simplex virus, cytomegalovirus virus), particularly in immunocompromised patients.^28^, ^29^ Other known triggers, including thiol drug use, infections, vitamin D deficiency, radiotherapy, pregnancy, trauma, stress, and vaccines, can influence time to relapse.^30^

### 3.3 Medications and associated relapse rates

#### 3.3.1 Corticosteroids

Corticosteroids are potent, anti‐inflammatory and immunosuppressive agents that downregulate inflammatory mediators, reducing B‐cell expansion and alleviating symptoms. Since their introduction in the 1950s, corticosteroids have reduced mortality rates among patients with pemphigus from 75% to 30%.^31^ High‐dose and low‐dose corticosteroids show no difference in relapse or remission rates at 5 years, although adjuvant therapy may decrease the risk of relapse by 29%.^32^, ^33^, ^34^

The relative rate of complete remission off‐therapy after 36–39 months was similar in different dosing cohorts: 14/32 (43.7%) in the medium dosing (≤0.5 mg/kg/day; *n* = 32) corticosteroid group, 31/59 (52.5%) in the high dose (≥1 mg/kg/day; *n* = 59) corticosteroid group, and 23/43 (53.5%) in the non‐corticosteroid group (*p* = 0.32).^35^ The impact of adjuvant therapy is varied; a randomized clinical trial (RCT) compared prednisolone alone with prednisolone plus azathioprine, mycophenolate mofetil, or cyclophosphamide and found relapse rates among these groups to be 30.4%, 21.7%, 28.6%, and 35.0%, respectively (*p* = 0.80).^36^

#### 3.3.2 Rituximab

Rituximab is an FDA‐approved CD20 inhibitor for moderate to severe PV, which functions by removing B‐cell clonal populations that produce autoreactive IgG4 antibodies to DSG1 and DSG3.

Two additional studies evaluating 1000 mg intravenous (IV) rituximab found low relapse rates (11%–16%).^26^, ^37^ Time until relapse from last dose of treatment varied greatly across studies, with median times ranging from 12.29 to 36 months.^38^ Relapse rates also increased with time (15.3% at 1 year and 39.1% at 2 years). Duration of remission was 11.45 months and ranged from 3 to 22 months following treatment in patients receiving 1 g IV for 1–3 months.^39^

In an evaluation of rituximab dosing regimens, relapse was more common in patients with low‐dose rituximab, suggestive that more effective B‐cell recruitment with higher doses may be linked to better rates of remission (Table 1).^54^ Patients achieved remission faster and time of remission was longer in rituximab‐treated groups. Low dose rituximab (500 mg weekly × 2 weeks) was associated with a higher relapse rate (64%) compared to high dose (1000 mg × 2 weeks) (36%).^41^

**TABLE 1:** Selected studies evaluating pemphigus outcomes following rituximab administration.

Author	Study type	Enrollment	Indication	Drug regimen	Relapse endpoints		
Joly et al.^40^	RCT	91 patients	Pemphigus	Prednisone 1–1.5 mg/kg over 12–18 months Or 1000 mg RTX Day 0, 14 and 500 mg at month 12 and 18 with short term prednisone 0.5–1.0 mg/kg tapered over 3–6 months	Median delay to complete remission off therapy: 677 days prednisone, and 277 Prednisone + RTX Median cumulative duration of complete remission: 446 days RTX vs 62 days prednisone only (*p* < 0.0001) Month 24: Relapse in 24% of RTX treatment and 45% prednisone 2‐year disease free survival 36.7% prednisone, 75.4% RTX (*p* = 0.0191) 3‐year follow up in patients complete remission by 24 months: 1 relapse RTX, and 4 prednisone		
Kanwar et al.^41^	RCT	22	Pemphigus	1000 mg RTX Day 0, 15 Or 500 mg RTX Day 0, 15	Time to disease control non‐significant (*p* = 0.49) Relapse: 4/11 (36%) patients high dose, 7/11 low dose (64%) (*p* = 0.20) Time to relapse: 36 weeks high dose, 32 weeks low dose (*p* = 0.29)		
Amber et al.^14^	Retrospective review	155	Pemphigus	1. RA protocol: 1000 mg weekly × 2 weeks Or 2. Half‐RA protocol: 500 mg weekly × 2 weeks Or 3. LP: 375 mg/m^2^ × 4 weeks Or 4. Half‐LP: 375 mg/m^2^ × 2 weeks	12‐month relapse free score	18‐month relapse free score	24‐month relapse‐free score
					1 vs 2: *p* < 0.001 4 vs 3: *p* = 0.31 1 vs 3: *p* = 0.28 2 vs 4: *p* < 0.01 2 vs 4 weeks: *p* = 0.02	1 vs 2: *p* = 0.01 4 vs 3: *p* = 0.43 1 vs 3: *p* = 0.33 2 vs 4: *p* = 0.02 2 vs 4 weeks: *p* = 0.04	1 versus 2: *p* = 0.03 4 versus 3: *p* = 0.55 1 versus 3: *p* = 0.31 2 versus 4: *p* = 0.04 2 versus 4 weeks: *p* < 0.05
Wang et al.^42^	Systematic review + meta‐ analysis	578 patients	Pemphigus	High dose (near or >2000 mg/cycle) vs Low dose (<1500 mg/cycle)	76% of patients achieved complete remission after 1 cycle of RTX 5.8 months until remission With 14.5‐month remission duration 40% relapse rate		
Singh et al.^43^	Prospective, randomized, non‐blinded parallel‐group pilot study	23	Severe pemphigus	Group A: 1000 mg two doses, 2 weeks apart Group B: 500 mg, two doses, 2 weeks apart	Time to complete remission of therapy: 27.1 weeks Group A vs 26 weeks Group B (*p* = 0.09) Immunological relapse %: 77% Group A vs 91% group B (*p* = 0.47) Time to immunological relapse: 8.2 months Group A vs 8.4 months Group B (*p* = 0.87)		
Saleh et al.^44^	RCT	17	Relapsing pemphigus patients	A: Full dose RTX (1000 mg IV/week × 2 weeks) B: Half dose RTX (1000 mg IV/week × 1 week)	6/8 (75%) in Group A achieved complete remission vs 1/9 (11.1%) in Group B (*p* < 0.0001) 80%–100% decrease in PDAI Group A vs 0%–100% decrease in PDAI Group B (*p =* 0.03)		
Das et al.^45^	Retrospective data analysis	12	Pemphigus	RA Protocol (Group 1): Infusion of 1 g (2 doses 15 days apart) Dexamethasone cyclophosphamide pulse protocol (Group 2): 100 mg of dexamethasone for 3 days plus 500 mg of cyclophosphamide on Day 2 to be repeated every 28th day along with oral cyclophosphamide 50 mg daily during the pulse‐free interval	Group 1 Relapse Rate: 0/6 (0%) Group 2 Relapse Rate: 2/6 (33.3%) Group 1 mean PAAS cutaneous score at baseline: 7.7 Group 1 mean PAAS cutaneous score at 9 months: 0 (*p* = <0.001) Group 2 mean PAAS cutaneous score at baseline: 6.96 Group 2 mean PAAS cutaneous score at 9 months: 0.66 (*p* = <0.001)		
Sharma et al.^46^	Retrospective data analysis	25	Pemphigus	Twenty‐one patients received two doses of 1 g of RTX, 2 weeks apart One patient received three doses of a 500 mg infusion at intervals of 2 weeks Two patients received four doses of a 500 mg infusion at intervals of 2 weeks One patient received four doses of 640 mg at intervals of 2 weeks	Complete Remission: 22/25 (88%) of patients Partial Remission: 3/25 (12%) of patients Mean time to disease control: 1.10 months Mean time to complete remission: 4.36 months 4/25 (16%) of patients experienced mild relapse after a mean duration of 11.75 months		
Mezni et al.^47^	Retrospective Analysis	43	Pemphigus	Two doses of 1 g of RTX, 15 days apart with patients divided into naive (RTX as a first‐line therapy) group and a non‐naive group (RTX as a subsequent therapy)	Relapse rate over a mean period of 16.27 months: 23.6% 83.7% of patients achieved complete remission by the third month There was no statistical difference (*p* > 0.05) between the naive and non‐naive group with regards to remission and relapse rates		
Anuntrangsee et al.^48^	Retrospective chart analysis	50	Pemphigus	Variable	40/50 patients (80%) achieved complete remission over a median time of 6.66 months 14/50 patients (35%) relapsed with median of 14.69 months after clinical remission Patients who received RT as a first‐line therapy were 2.45× more likely to achieve complete remission on therapy (*p* = 0.01)		
Simpson et al.^49^	Retrospective chart analysis	8	Pemphigus	Two infusions of 200 mg RTX, 14 days apart	Relapse rate: 25% Time until relapse ranged 184–386 days		
Parajuli et al.^37^	Retrospective analysis	9	Pemphigus	1 g IV RTX on Day one and Day 15 with additional 500 mg IV on 12th and 18th months combined with Prednisolone (1 mg/kg/day) gradually tapered over 3–6 months	1/9 (11%) of patients experienced relapse Complete remission: 7/9 patients (77.7%)		
Sakhiya et al.^50^	Retrospective chart analysis	32	Pemphigus	2–5 infusions of 1000 mg IV weekly over 4 weeks	5/32 (15.6%) relapsed. Median time until relapse 96 weeks Anti‐DSG3 antibody levels were high in all patients who relapsed		
Chen et al.^51^	Retrospective analysis	74 patients	Pemphigus	LP: 375 mg/m^2^ RA protocol: 1000 mg IV Day 1, Day15	Complete remission rate: 66.2% Median time until complete remission: 10.95 months Relapse rate in complete responders: 27.1% Median time until relapse: 12.29 months 1‐year relapse rate: 15.3% 2‐year relapse rate: 39.1%		
Perifani et al.^52^	Prospective observational	25	Refractory pemphigus	375 mg/m^2^ IV weekly × 4 weeks	11/25 (44%) of patients relapsed Median PDAI at relapse was 25		
Laftah et al.^38^	10‐year follow‐up study	10	Pemphigus vulgaris	375 mg/m^2^ weekly × 8 weeks then once monthly for × 4 months	At 10‐year follow‐up, 44% of patients relapsed Mean time to relapse after one course was 36 months		
Verma et al.^26^	Retrospective study	17	Pemphigus	1000 mg on Day 1 and Day 15	2/11 (18.1%) patients that had complete remission relapsed after 18 months of follow‐up 2/4 (50%) patients below 20 years old relapsed at 18 months		
Bilgic‐Temel et al.^27^	Retrospective analysis	5	Recalcitrant pediatric pemphigus	RTX 500–1000 mg Day 1 and Day 15 Or 375 mg/m^2^ once weekly × 4 weeks	40% relapse rate		
Anandan et al.^39^	Open label prospective interventional study	20	Pemphigus	1 g IV every 2 weeks for 1–3 months	Duration of remission ranged from 3 to 22 months (mean 11.45 months) 35% of patients relapsed (2 at 6 months, 3 at 10 months, 2 at 16 months)		
Kura et al.^53^	Prospective interventional study	15	Pemphigus and bullous pemphigoid	375 mg/m^2^ weekly × 4 weeks	93% of patients had complete remission 4/15 (27%) relapsed at 3, 5, 8, and 10 months respectively		

Abbreviations: IV, intravenous; LP, lymphoma protocol; PAAS, pemphigus area, and activity score; PDAI, pemphigus disease area index; RA, rheumatoid arthritis; RCT, randomized controlled trial; RTX, rituximab.

Similarly, another study found that patients receiving high‐dose rituximab (>2000 mg/cycle) had higher rates of complete remission compared to low‐dose rituximab (<1500 mg).^42^ Other studies had conflicting results, with one study showing no clinical difference in time until remission and relapse rates in high versus low dose rituximab regimens.^43^

Low‐dose rituximab relapse rates were still lower than the standard half LP (375 mg/m^2^ × 4 weeks) at 12, 18, and 24 months, though 4‐week dosing protocols had better relapse‐free scores than 2‐week regimens.^14^ However, low‐dose rituximab is relatively safe and efficacious for patients with mild disease with a 25% relapse rate (range 184–386 days).^49^

Patients who received rituximab dosing protocols that were weight‐based and lower in frequency tended to have higher relapse rates (27%–45%).^27^, ^52^ Recalcitrant pediatric pemphigus patients had a 40% relapse rate.^27^

In one study, after treatment with IV rituximab, 88% of patients achieved complete remission with four (16%) patients experiencing relapse at a mean duration of 11.25 months, whereas another study found that 83.7% of patients achieved complete remission within 3 months with a relapse rate of 23.6% and a mean of 16.27 months until relapse.^46^ Overall, patients who received rituximab as a first‐line treatment had a 2.45 times greater likelihood of achieving complete remission on therapy, with a 35% relapse rate at a median of 14.69 months.^48^

In patients that experience relapse, retreatment with high dose rituximab (1000 mg/week × 2 weeks) is associated with lower relapse rates and greater reductions in PDAI (*p* < 0.001 and *p* = 0.03 respectively) than with a half‐cycle dose (1000 mg/1 week × 1 week).^44^

In summary, high dose rituximab regimens, including 1000 mg/week × 2 weeks and >2000 mg/cycle, have been shown to be effective in preventing pemphigus relapse.

#### 3.3.3 Mycophenolate mofetil

Mycophenolate mofetil is an immunosuppressive adjuvant used in conjunction with corticosteroids for the treatment of PV.^31^ In a previous RCT studying mycophenolate mofetil (2 or 3 g/day) plus oral prednisone, both treatments were found to have similar efficacy (*p* = 0.66) compared to prednisone only; however, the combination had a lower relapse rate (21.8%) compared to placebo (44.5%, *p* = 0.0343) at 24 weeks.^55^

Mycophenolate mofetil has a significantly shorter time to achieve complete remission on therapy and requires significantly reduced steroid doses compared to azathioprine. A study examining both found that in the azathioprine group, 4/30 patients had recurrence after achieving the end of the consolidation phase compared to 1/15 patients in the mycophenolate mofetil group (Table 2).^56^ An RCT comparing rituximab IV infusion plus mycophenolate mofetil placebo with mycophenolate mofetil plus rituximab IV placebo found that patients in the rituximab group had fewer pemphigus flares (9.7% vs 69.8%, p  < 0.0001).^57^

**TABLE 2:** Selected studies evaluating pemphigus outcomes following mycophenolate mofetil administration.

Author	Study type	Enrollment	Indication	Drug regimen	Relapse endpoints
Sukanjanapong et al.^56^	Retrospective Cohort	62	Pemphigus	Prednisolone + MMF Or Prednisolone + AZA	Complete remission on therapy: AZA 73%; MMF 72% *p* = 0.933 Median time to reach complete remission on therapy: AZA 12.5 months; MMF 7.3 months *p* = 0.019 Complete remission off therapy: AZA 24.3%; MMF 20% *p* = 0.690 Cumulative steroid dose required to achieve complete remission on therapy: AZA 7740 mg; MMF 3473.8 mg *p* = 0.007 Relapse rate: AZA 4/30 (13.3%); MMF 1/15 (6.67%)
Werth et al.^57^	RCT	135	Pemphigus	Rituximab IV Infusion + MMF placebo Or MMF BID + Rituximab IV infusion placebo	Proportion of patients achieving complete remission (defined by PDAI as the appearance of 3+ lesions in a patient who had previously achieved disease control): Rituximab 6%; MMF 44% *p <* 0.0001 Total number of disease flares: Rituximab 6/62 (9.7%); MMF 44/63 (69.8%) *p <* 0.0001

Abbreviations: AZA, azathioprine; BID, two times a day; MMF, mycophenolate mofetil; RCT, randomized controlled trial.

#### 3.3.4 Azathioprine

Azathioprine is another steroid‐sparing, immunosuppressive adjuvant used in conjunction with corticosteroids for the treatment of PV. An RCT comparing prednisolone plus placebo with prednisolone plus azathioprine found that 82.4% of patients in the azathioprine group achieved complete remission compared to 41.2% in the placebo group.^58^ The azathioprine group had a lower relapse rate (39.3%) compared to the corticosteroid group (64.3%).^58^

A long‐term study evaluated the effectiveness of azathioprine as an adjuvant therapy for oral PV. All patients were initially treated with oral prednisolone (1 mg/kg/day for 4–6 weeks). There was no difference in the number of patients achieving complete remission during treatment (*p* = 0.645), the number of patients who relapsed once (44% with oral prednisolone alone and 54.5% with azathioprine, *p* = 0.108), or the time to achieve complete remission (*p* = 0.343).^59^

#### 3.3.5 Cyclophosphamide

Cyclophosphamide is a potent immunosuppressive medication that inhibits DNA synthesis and affects the proliferation of B and T lymphocytes, reducing the production of autoantibodies responsible for blister formation in PV. Cyclophosphamide is used as part of pulse therapy, which typically involves administering higher doses of the drug at specific intervals (e.g., once a month or in alternating weeks) rather than a continuous daily dosage. This intermittent dosing regimen can help reduce the cumulative toxicity associated with the medication while still providing its therapeutic effects.

A trial investigating cyclophosphamide pulse therapy (CPT) (15 mg/kg once a month for a year as an adjunct with corticosteroids) found that corticosteroid monotherapy had 71% relapse on treatment versus 51.8% relapse with combination therapy (*p* = 0.9) (Table 3). About 60% of patients on corticosteroid monotherapy relapsed after a median of 9 weeks post‐treatment, while 42.3% of patients on combination therapy relapsed after a median of 4 weeks post‐treatment (*p* = 0.53).^60^ Historically, dexamethasone pulse therapy (DCP) in combination with daily oral cyclophosphamide was found to have comparable relapse rates to CPT with tapering doses of oral prednisone in a study involving 28 PV patients (62.5% vs 66.6% at mean 14 and 12.6 weeks after stopping treatment respectively) *p* = 1.0.^64^

**TABLE 3:** Selected studies evaluating pemphigus outcomes following cyclophosphamide administration.

Author	Study type	Enrollment	Indication	Drug regimen	Relapse endpoints
Sharma & Khandpur^60^	Randomized, prospective, non‐blinded trial	60	Pemphigus vulgaris	Daily oral prednisone vs IV CPT + prednisolone for 1 year	71% vs 51.8% relapse on treatment (*p* = 0.9) 60% vs 42.3% relapse at median 9‐ and 4‐weeks post‐treatment, respectively (*p* = 0.53)
Parmar et al.^61^	Preliminary prospective RCT	19	Pemphigus vulgaris	Phase I (all patients): monthly DCP Phase II: Group A (10 patients) received monthly DCP for 9 months, Group B (9 patients) received oral cyclophosphamide for 9 months	1/10 (10%) vs 1/9 (11.1%) relapse by 9 months
Hassan et al.^62^	Retrospective and prospective non‐comparative study	47	Pemphigus	DCP therapy vs DAP therapy vs DMP therapy	DCP: 3/30 (10%) relapsed during phase IV. DAP: 5/12 (41.7%) relapsed in phase III and 4/12 (33.3%) vs relapsed in phase IV DMP: discontinued due to poor disease control
Zeeshan et al.^63^	5‐year retrospective study	102	Pemphigus	DCP therapy	15/72 (20.8%) relapse rate

Abbreviations: CPT, cyclophosphamide pulse therapy; DAP, dexamethasone‐azathioprine pulse; DCP, dexamethasone‐cyclophosphamide pulse; DMP, dexamethasone‐methotrexate pulse; IV, intravenous; RCT, randomized controlled trial.

In a non‐comparative study evaluating DCP versus dexamethasone azathioprine pulse (DAP) versus dexamethasone‐methotrexate pulse (DMP), 3/30 (10%) patients given DCP relapsed during phase IV of the study. Of 12 patients given DAP therapy, five patients relapsed in phase III and four patients relapsed in phase IV. The DMP arm was discontinued because of poor disease control.^62^ In a preliminary prospective, RCT where 10 patients received monthly DCP therapy for 9 months while nine patients received only oral cyclophosphamide for 9 months, it was found that one patient in each group experienced relapse by the end of 9 months (10% vs 11.1%).^61^

The long‐term outlook with cyclophosphamide is also promising. A 5‐year retrospective study involving 102 pemphigus patients on DCP therapy found that of 72 patients who achieved remission, a total of 15 relapsed (20.8%), with 11 of these relapses resulting from irregular or discontinued treatment.^63^

#### 3.3.6 IV immunoglobulin

Intravenous immunoglobulin is an adjuvant, second‐line therapy for pemphigus that consists of pooled human plasma with >95% IgG antibodies and a small amount of IgM and IgA.^1^ A retrospective analysis of 123 pemphigus patients on a multidrug protocol–a short course of prednisone and long‐term maintenance with IVIg in addition to a cytotoxic immunosuppressive drug, a tetracycline derivative, and nicotinamide–showed >5‐year stable remission in 88% of patients.^20^, ^65^ Additionally, a recent study examining 105 pemphigus foliaceus (PF) patients across 41 studies, treated with rituximab, IVIg, or a combination of the two, found that the IVIg group had complete remission in 62.5% (5/8) of patients, with no relapses or infections at mean follow‐up of 24.8 months.^66^ In a study involving 30 pemphigus patients across these same treatment groups, 23 patients received the combination of rituximab and IVIg, six patients received rituximab alone, and one patient received IVIg alone; 11 patients relapsed.^67^ In a single‐center case series involving 63 pemphigus patients, 23 patients with refractory PV or PF received IVIg, and 15 of these patients did not eventually require rituximab, while 14 of these patients achieved clinical remission or partial remission after an average of 3.8 months. Clinical remission was more likely in patients treated with IVIg or rituximab compared to those treated with systemic corticosteroids (*p* = 0.000467). Serologic remission was more likely in patients treated with systemic corticosteroids or rituximab (*p* = 0.002118).^68^

#### 3.3.7 Dapsone

Dapsone is another second‐line, steroid‐sparing adjuvant therapy that yields anti‐inflammatory effects via the suppression of tumor necrosis factor‐α and interleukin‐8, reducing B‐cell proliferation. In a case series involving patients who were resistant or intolerant to rituximab, dapsone was evaluated in combination with other adjuvants. In one patient taking MMF, methotrexate, and dapsone, the prednisone dose was reduced from 80 mg at onset to 10 mg at complete remission. The other two patients in the study who were treated with dapsone did not achieve complete remission. Prior to the use of immunosuppressants including dapsone, the three patients experienced five, seven, and one relapses respectively, which was reduced to three, two, and zero relapses with the use of adjuvants including dapsone. Combination therapy reduced the number of relapses in treatment‐resistant patients, however, the impact of dapsone monotherapy is unknown.^69^

#### 3.3.8 Ofatumumab

Ofatumumab is a B‐cell depleting monoclonal antibody that targets CD20, thereby reducing the production of autoantibodies.^70^ A potential advantage of ofatumumab over rituximab is that it originates from a human antibody which may translate to less immunogenicity, its subcutaneous administration, and its potential use in pregnancy.^71^ A clinical trial comparing ofatumumab with placebo in 35 pemphigus patients found that after 60 weeks of treatment, the group of participants receiving ofatumumab had a 47% relapse compared to 77% in the placebo group, with an average of 448 days until relapse compared to 169 days until relapse in the placebo group.^72^

#### 3.3.9 Rilzabrutinib

Rilzabrutinib works by inhibiting Bruton's tyrosine kinase, an enzyme involved in B‐cell signaling and activation ultimately reducing the production of autoantibodies, which may be clinically efficacious in PV. A recent clinical trial in 2021 followed 27 PV patients who were given rilzabrutinib for treatment; 52% of those patients showed control of disease activity after 12 weeks, with two patients relapsing at week 20.^73^ Another clinical trial evaluated rilzabrutinib in 131 pemphigus patients compared with the use of placebo and found that 60% of pemphigus patients experienced relapse after 37 weeks compared to 30% who used rilzabrutinib.^74^ Unfortunately, a Phase III RCT evaluating rilzabrutinib for moderate/severe PV was discontinued due to lack of efficacy.

## 4 DISCUSSION

Relapse occurs in greater than 50% of pemphigus patients. Identification of risk factors and relapse rates associated with different agents may guide therapeutic selection.^6^ Rituximab revolutionized treatment and became the first FDA‐approved medication for moderate to severe PV. It can induce complete remission in 50% to 90% of pemphigus patients; however, relapse generally occurs between 6 and 26 months.^44^ Furthermore, there is an important consideration for PV patients receiving treatment early in the clinical course versus patients being treated after multiple relapses as those with refractory disease may have comorbidities or diminished compliance with treatment, which may increase relapse potential.^33^ Additionally, patients with severe pemphigus are more likely to relapse earlier in their clinical course.^33^ Some patients may have lifelong remission from pemphigus, which may occur as a result of the complete eradication of responsible B‐cell populations. Future targeted therapies should aim at selective identification and eradication of errant B‐cell populations that are producing autoantibodies.

In most patients who experience relapse it is due to incomplete penetrance of B‐cell depletion on causative B‐cell populations and/or CD20‐ B‐cell populations that reactivate after time or stimulation. Risk factors for pemphigus relapse include patients with high PDAI at onset, high BMI, high affected BSA, non‐mucosal pemphigus subtypes, PV, and high DSG1 and DSG3 populations after treatment. The mean baseline anti‐DSG1 index is different between patients with early relapse versus late relapse (*p* = 0.0014), and there is a negative correlation between baseline DSG1 index and time to relapse (*p* = 0.00009).^63^ Although these risk factors have been identified, there are limited studies evaluating targeted interventions in treatment and prevention of these risk factors and associations with clinical relapse. Additionally, patients who receive rituximab rheumatoid arthritis dosing with higher doses and frequency as well as those who receive earlier treatment have better clinical endpoints and lower rates of disease relapse.^1^, ^2^, ^75^, ^76^, ^77^

Pemphigus‐related conditions are associated with significant diagnostic delay, including a mean of 6.19 ± 3.82 months' time delay until diagnosis.^78^ Targeted modalities to improve a timely diagnosis, including improved health literacy, increased awareness across patients and healthcare providers, and improved access to care, may improve time to treatment and, subsequently, lower relapse rates. The majority of patients who relapse from pemphigus receive non‐rituximab treatments as well as delayed treatment with sub‐therapeutic treatment dosages for shorter periods.^78^ The mean time of B‐cell repopulation is impacted by interindividual variability but averages 6–9 months.^79^ Other factors that may heighten relapse risk and decrease therapeutic response to CD20+ inhibitors include B‐cell activating factor /inflammatory stimuli lowering serum anti‐CD20 medication concentration and production of anti‐drug antibodies (present in up to 56% of patients).^44^, ^80^ Genetic polymorphisms, such as FCGR3A F158V, are associated with improved responsiveness to rituximab, whereas FCGR2A R131H was found not to be associated with a better response.^81^ Despite known drug‐gene interactions, the clinical relevance and implications of treatment selection/course of care have not been evaluated.

Although rituximab therapy is considered the gold standard of therapy, the optimal dosing strategy is unclear. In this review, we found inconsistencies between clinical outcomes utilizing lymphoma and rheumatoid arthritis‐based dosing protocols.^23^, ^42^, ^43^ However, regardless of dosing strategy, more frequent dosing with larger cumulative dosing was associated with lower relapse potential, which is likely due to increased drug accumulation and B‐cell depletion. Adversely, rituximab is associated with potentially life‐threatening, infusion‐related reactions, heightened infection risk, and osteomyelitis risk, although the risk of death due to pemphigus and cardiovascular causes was much lower.^31^ These risks may be more prevalent with higher doses, increased number of cycles of treatment, and increased repeated exposures. Alternative agents, such as mycophenolate mofetil and azathioprine, have shown clinical utility; however, the lack of large‐scale, robust clinical studies accompanied with complications of long‐term use (AE risks, tolerability, etc.) limit their use clinically.

There are some limitations to this review. Differences in treatment regimens including exact dose, duration, and frequency across low sample sizes make cross‐comparisons and identification of optimal dosing schedules challenging. Relapse rates were also evaluated at set time markers in some studies and were not standardized across studies. This may underestimate the true time until relapse of some patients, and relapses occurring after the study treatment may not have been collected. Variances in study participants may also complicate the generalizability of study findings. Additionally, many studies were retrospective and case studies with limited clinical trials with large cohorts evaluated, which is likely to reflect the rarity of pemphigus conditions. Future studies should include large comparator clinical trials to evaluate relapse rates for multiple treatment modalities.

## 5 CONCLUSION

The clinical course of pemphigus is heterogeneous: patients experience varying periods of relapse‐free disease remission and then periods of relapse occurring anywhere between months to many years after treatment. Mechanisms of relapse include incomplete B‐cell depletion, de novo synthesis of antibody‐producing B‐cells, and persistence of CD20 long‐lived B‐cells, among others.

The current standard of care for moderate to severe pemphigus is rituximab and adjuvant corticosteroid treatment, with associated relapse rates ranging from 11%–40% in the studies evaluated. Higher rates of relapse and longer time until disease control was associated with lower and less frequent rituximab dosing strategies. Diagnostic delay is common in pemphigus, and gaps in time from symptoms onset to diagnosis and treatment further increase relapse risk. Other therapeutic alternatives including mycophenolate mofetil, azathioprine, IVIg, and rilzabrutinib have shown clinical efficacy but are associated with higher relapse risk and studies evaluating their potential have been conducted on a smaller scale.

## CONFLICT OF INTEREST STATEMENT

None declared
